# Gender-related asymmetric brain vasomotor response to color stimulation: a functional transcranial Doppler spectroscopy study

**DOI:** 10.1186/2040-7378-2-21

**Published:** 2010-11-30

**Authors:** Philip C Njemanze

**Affiliations:** 1 Non-invasive Neurocybernetic Flow Laboratory, International Institutes of Advanced Research and raining, Chidicon Medical Center, No 1 MCC/Uratta Road, P.O. Box 302, Owerri, Imo State, 460242, Nigeria

## Abstract

**Background and Purpose:**

The present study was designed to examine the effects of color stimulation on cerebral blood mean flow velocity (MFV) in men and women.

**Methods:**

The study included 16 (8 men and 8 women) right-handed healthy subjects. The MFV was recorded simultaneously in both right and left middle cerebral arteries in Dark and white Light conditions, and during color (Blue, Yellow and Red) stimulations, and was analyzed using functional transcranial Doppler spectroscopy (*f*TCDS) technique.

**Results:**

Color processing occurred within cortico-subcortical circuits. In men, wavelength-differencing of Yellow/Blue pairs occurred within the right hemisphere by processes of cortical long-term depression (CLTD) and subcortical long-term potentiation (SLTP). Conversely, in women, frequency-differencing of Blue/Yellow pairs occurred within the left hemisphere by processes of cortical long-term potentiation (CLTP) and subcortical long-term depression (SLTD). In both genders, there was luminance effect in the left hemisphere, while in men it was along an axis opposite (orthogonal) to that of chromatic effect, in women, it was parallel.

**Conclusion:**

Gender-related differences in color processing demonstrated a right hemisphere cognitive style for wavelength-differencing in men, and a left hemisphere cognitive style for frequency-differencing in women. There are potential applications of *f*TCDS technique, for stroke rehabilitation and monitoring of drug effects.

## Introduction

Conventional neuroimaging techniques have not been applied to resolve the dual effects of light both as a wave and as quanta energy, on the visual system. Hence the neuroadaptation to the physical qualities of light and their role in mechanisms for color processing, remains unknown. It could be presumed that, the neuronal assemblies processing color information and their blood flow supply vascular network, share analogous topological and functional organization [1]. Therefore, during 'functional coupling' of the neuronal and vascular compartments, there is relatedness and hence universality of frequency responses in both systems. It would be expected that the neuronal activity would elicit proportional frequency responses in vasomotor activity. The resulting oscillatory components of the cerebrovascular system satisfy two basic postulates namely periodicity and linearity, which are prerequisite conditions for application of Fourier analysis [2,3].

Fourier analysis could be applied to decompose the complex pulse wave oscillations into a series of sine waves characterized by amplitude, frequency and phase shifts [4,5]. The fundamental frequency is the frequency of the first sine wave or first harmonic, which for example, in a person with heart rate of 60 beats per minute, would be 1 Hz. The second harmonic is twice the frequency of the first, and so on. The first five harmonics contain about 95% of the pulsatile energy from left ventricular contraction [6]. Fourier analysis has been widely applied for resolving bioelectric waveforms into their various frequency constituents, described as spectral electroencephalography [7], high resolution electrocardiography [8] and high resolution transcranial Doppler [3]. Recently, Fourier analysis was applied to analyze waveform envelops comprising several cycles of mean flow velocity (MFV) fluctuations recorded in the right (RMCA) and left (LMCA) middle cerebral arteries during visual stimulations, and has been described as functional transcranial Doppler spectroscopy (*f*TCDS) [9,10]. The *f*TCDS technique differentiates signals emanating from the cortical and subcortical branches of the cerebral arteries of the circle of Willis [9]. The frequencies with the greatest spectral density estimates were designated as spectral peaks deriving from reflection sites at the terminal cortical (**C**-peak) and subcortical (**S**-peak) sites within the MCA territory. The vasomotor oscillations indexed by *f*TCDS during color stimulations in simulated microgravity head-down rest positions have demonstrated characteristics of long-term potentiation (LTP) and long-term depression (LTD), at cortical (CLTP and CLTD) and subcortical (SLTP and SLTD) peaks lasting several hours [10], similar to expected summation of cellular LTP [11] and LTD [12] phenomena.

Recently, it was demonstrated using *f*TCDS and functional transcranial Doppler (*f*TCD) that, while men employed a right hemisphere cognitive style, women implemented a left hemisphere cognitive style, during facial [9,13] and general intelligence processing [14]. The latter findings have been confirmed using other modalities [15-17]. It was therefore hypothesized that, color processing may follow similar rules of lateralization [18,19]. The present study was designed to examine the effects of color stimulation on MFV in the RMCA and LMCA in men and women, respectively. It was expected that, there would be gender-related differences in color processing. Men would implement right hemisphere wavelength-differencing, while women would employ left hemisphere frequency-differencing. The gender-related hemisphere asymmetry, may be as a result of neuroadaptation to the physical quality of light as a wave and as quanta energy.

## Subjects and Methods

The study included 8 men of mean ± SD age of 24.6 ± 2 years, and 8 women of mean ± SD age of 24 ± 2 years. All subjects were right handed as determined using a hand preference questionnaire [20]. The participants had normal visual acuity tested using Snellen chart, normal color vision on tests with Ishihara pseudoisochromatic plates and normal color recognition [21]. The vital signs including pulse rate, blood pressure, and respiratory rate were within normal limits. The neurologic examine was unremarkable. Self-perceived anxiety levels using a state-trait anxiety inventory (STAI) in pre-test and post test conditions were also normal [22]. All participants abstained from use of caffeine, nicotine and any medications for at least 72 hours preceding testing [23]. Written informed consent was obtained from all subjects according to the Declaration of Helsinki; and the Institutional Ethics Board approved the study protocol. The experimental procedure including transcranial Doppler (TCD) scanning was similar to that used in prior cognitive studies described in detail elsewhere [10,19]. In summary, TCD studies were performed using two 2 MHz probes of a bilateral simultaneous TCD instrument (Multi-Dop T, DWL, Singen, Germany), with sample volume placed in the RMCA and LMCA main stems at a depth of 50 mm. The recordings were performed with the subject lying supine, with head and trunk elevated at 30 degrees.

### Tasks

The tasks were designed in our laboratory, and have demonstrated consistency and reliability with TCD ultrasonography in prior studies described in detail elsewhere [10,19]. In summary, a specially adapted 3D-viewing device (Viewmaster, Portland, OR) was painted inside with black paint. The right aperture of the device was closed to light, but the left aperture was open, to be backlit from white light reflected from a remotely placed light source. The light source was projected onto a white surface flat screen, placed 125 cm from the lamp. The screen was positioned 80 cm from the nose ridge of the subject. The light source was a tungsten coil filament, of a general service lamp ran at a constant rating of 24 V and 200 W, with a color temperature of about 2980 K and approximately 20 lumens/watt. Optical homogenous filters were placed on the reel of the Viewmaster, in the light path for color stimulation. Kodak Wratten filters: Deep Blue (No. 47B) with short dominant wavelength (λ) of ***S***_λ _= 452.7 nm; Deep Yellow (No. 12) with medium dominant wavelength (λ) of ***M***_λ _= 510.7 nm, and Red Tricolour (No. 25) with long dominant wavelength (λ) of ***L***_λ _= 617.2 nm, were used. The manufacturer's manual provides the excitation purity and luminous transmittance for each filter [24].

### Visual Stimulation and Recording

Measurements in Dark were considered as stimulus absent condition, as well as including background effects of scotopic vision [18,25]. A continuous train of velocity waveform envelops, was recorded for 60 seconds, simultaneously, for the RMCA and LMCA, respectively, for each stimulus condition. In the baseline Dark resting state condition, the subject was mute, still, attention was focused and eyes fixed at the centre within the dark visual field, with no mental or manual tasks to perform. An observer monitored the subject for movement artefacts, which were marked and removed from recordings. Electrophysiological monitoring of pulse and respiration rates was recorded. During color stimulation, the condition was identical to that of baseline, except for use of slides that were Blue, Yellow, and Red. During measurements, the left aperture on the reel was open to light reflected from the remote light source, and was considered as Light condition. The closure of the right aperture was used to maximize stimulation of the left and right sides of the left eye that projects to the ocular dominance columns in both hemispheres and precluded binocular interaction due to stereopsis [26,27]. Velocity waveform envelops for the relevant 60 seconds intervals were first averaged in 10 seconds segments, to produce six values for each measurement condition. A total of 960 MFV data points in all subjects were used for further calculations.

### Fourier Analysis of MFV

The application of Fourier analysis to MFV data for hemodynamic and cognitive studies, including advantages and potential problems have been discussed in detail elsewhere [9,10]. Single series Fourier analysis (Time series and forecasting module, Statistica for Macintosh, StatSoft, OK), was applied to a dataset of 48 data points of MFV for each artery under each condition, in eight men and eight women, respectively. The frequencies with the greatest spectral densities; that is, the frequency regions, consisting of many adjacent frequencies, that contribute most to the overall periodic behaviour of the series for each artery, RMCA and LMCA, respectively, were identified. The peaks were plotted and designated as fundamental (**F**-peak), cortical (**C**-peak) and subcortical (**S**-peak). The **C**-peak and **S**-peak, were relevant for assessment of cortical and subcortical responses. The fundamental **F**-peak which originates from peripheral reflections was not used. The *f*TCDS technique has been successfully applied to analysis of facial [9] and color processing [10].

### Other Statistics

Statistical analyses were performed using the software package (Statistica, StatSoft, OK, USA). The main results were given as mean/SE/1.96*SE. The box and whiskers plots were used to display the numerical data through their five-number summary of the distribution of the observations and to evaluate the impact of outliers. To examine the presence of stimulus effect, the multivariant analyses of variance (MANOVA) with repeated measures was applied to the MFV data set, followed by planned Scheffé contrast that compared stimulus response relative to stimulus-absent Dark condition. An analysis of covariance (ANCOVA) with repeated measures was performed to demonstrate that the difference found during visual stimulation persists even when the differences in baseline condition were partialled out. Furthermore, one-way ANOVA of paired groups was used to assess differences in spectral density estimates between two minima including the peak (as maxima), under different stimulation conditions, for the RMCA and LMCA, respectively. The LUMINANCE effect was derived by comparison of Dark versus Light conditions, and the direction relative to chromatic axis was either opposite (orthogonal axis) or parallel axis. WAVELENGTH-encoding was present when the effects of longer wavelength color (Yellow) were accentuated over shorter wavelength color (Blue) [10]. Conversely, ENERGY-encoding was present when the effects of higher frequency color (Blue), was accentuated over lower frequency color (Yellow) [10]. WAVELENGTH-differencing implicated WAVELENGTH-encoding main effect at **S**-peaks, and at least a tendency for ENERGY-encoding at **C**-peaks [10]. WAVELENGTH-differencing requires that a chromatic contrast detector subserving one area of chromatic space, excite a chromatic detector of opposite type and/or inhibit a chromatic detector of the same type in neighbouring areas of chromatic space [26]. FREQUENCY-differencing involved ENERGY-encoding main effect at **C**-peaks, and at least a tendency at **S**-peaks. CLTP process accentuated **C**-peaks over **S**-peaks due to prevailing SLTD. Conversely, SLTP process accentuated **S**-peaks over **C**-peaks, due to prevailing CLTD.

## Results

**Figure 1:**
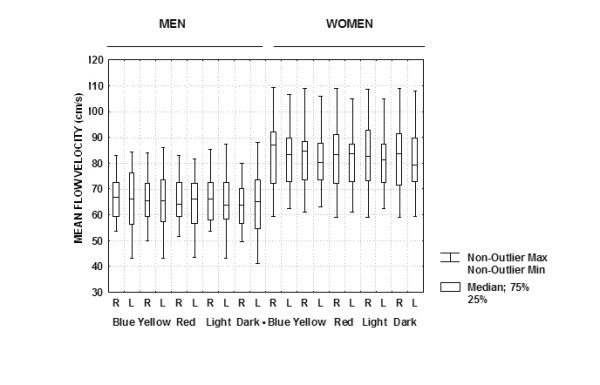
D**isplays the box and whiskers plots of the MFV data obtained for all subjects under all conditions of testing**.

Figure 1 displays the box and whiskers plot of the MFV data obtained for all subjects under all conditions. The five-number summary (the minimum, first quartile, median, third quartile, and maximum) of the distribution of the observations of the MFV data set showed a more symmetric distribution during color stimulations (Blue, Yellow, and Red) in men, compared to greater dispersion in women. Table 1 shows the mean ± SE of MFV for men and women, respectively, and the planned contrast (Scheffé *P*-value) variation from Dark condition. Overall, MFV for women were higher than that for men. In men, the RMCA MFV increased significantly in response to Light (2.6%), Blue (4.3%), Yellow (2.5%) and Red (2.8%). While LMCA MFV increased only to Blue (2.6%) stimulation. In women, only Blue stimulation evoked increase in MFV in the RMCA (2.6%) and LMCA (2.2%).

**Table 1:** Mean ± SE and Planned Contrasts of MFV Changes during Visual Stimulations from Dark Baseline in Men and Women

Stimulations	RMCA (cm/s)	Scheffé *P*	LMCA (cm/s)	Scheffé *P*
Men				
Dark	64 ± 1.26	-	63.9 ± 1.8	-
Light	65.7 ± 1.2	*< 0.05*	64.2 ± 1.67	*NS*
Blue	66.8 ± 1.28	<*0.0001*	65.6 ± 1.8	<*0.01*
Yellow	65.6 ± 1.26	<*0.05*	64.4 ± 1.7	*NS*
Red	65.8 ± 1.27	<*0.01*	64.4 ± 1.6	*NS*
Women				

Dark	81.6 ± 2	-	80.7 ± 1.8	-
Light	82.3 ± 2	*NS*	81.26 ± 1.7	*NS*
Blue	83.8 ± 1.9	<*0.0001*	81.6 ± 1.77	<*0.01*
Yellow	82.57 ± 1.86	*NS*	81.3 ± 1.6	*NS*
Red	82.1 ± 1.9	*NS*	80.7 ± 1.68	*NS*

The MANOVA was applied to MFV data set to assess differences between measurements in the RMCA and LMCA in men and women, with a 2 × 5 × 2 design: two levels of GENDER (Men and Women), five levels of STIMULATIONS, (Dark, Light, Blue, Yellow, and Red,), and two levels of ARTERY (RMCA and LMCA). The MFV was analyzed as the dependent variable. There was a main effect of GENDER, *F*(1,94) = 65.4, *MSE *= 68166, *P *< 0.0001. There was a main effect of STIMULATIONS, *F*(4,376) = 5.6, *MSE *= 111.7, *P *< 0.001. There was no main effect of ARTERY, *P *= NS. However, there was STIMULATION × ARTERY interaction, *F*(4,376) = 3.3, *MSE *= 5.96, *P *< 0.05.

**Figure 2:**
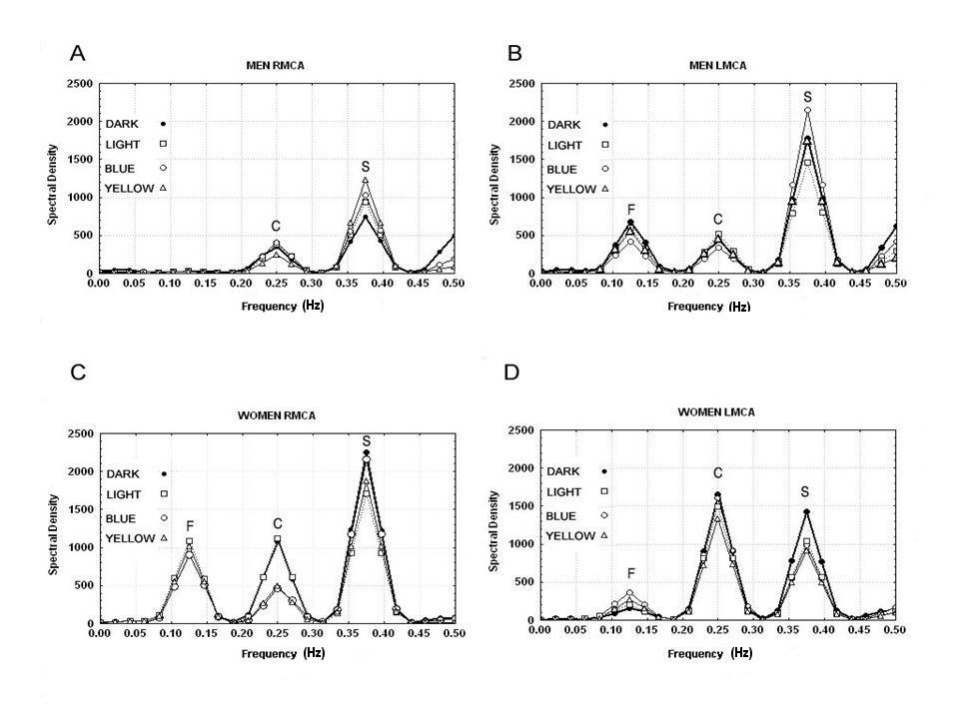
**(A-D) displays the conventional overlaid spectral density plots for each artery during stimulation conditions (Dark, Light, Blue and Yellow) in men (Figure 2A-B) and women (Figure 2C-D), respectively**.

Figures 2(A-D) demonstrates the conventional spectral density plots for each artery during Dark, Light, Blue and Yellow stimulations in men (Figures 2A-B) and women (Figures 2C-D), respectively. In general, for all stimulations in both men and women there were three peaks designated as fundamental (**F**-peak), cortical (**C**-peak), and subcortical **(S**-peak), which occurred at regular frequency intervals of the first (0.125 Hz), second (0.25 Hz), and third (0.375 Hz) harmonics, respectively. Given the differential MFV response in men and women, the spectral density estimates for men and women were analyzed separately, to uncover changes at cortical and subcortical peaks.

**Figure 3:**
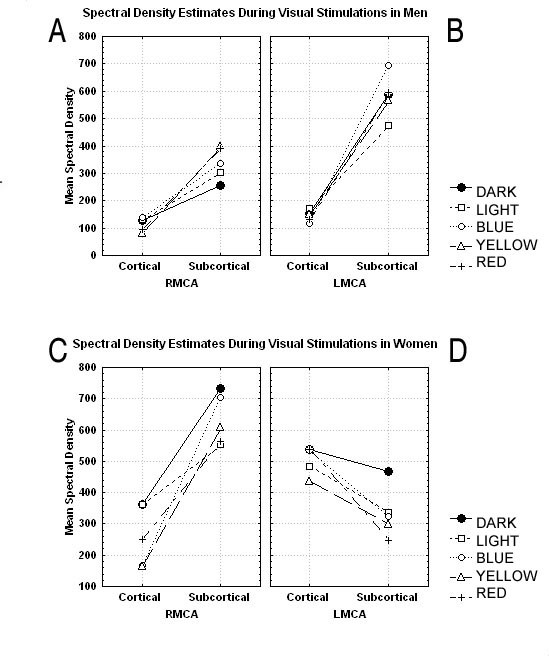
**(A-D) shows the plot of means of the three-way interaction of the spectral density estimates for all study conditions at C-peaks and S-peaks in men (Figure 3A-B), and women (Figure 3C-D), respectively**.

A MANOVA with repeated measures was applied to spectral density estimates in a 2 × 5 × 2 design: two levels of REGIONS (Cortical, Subcortical), five levels of STIMULATIONS (Dark, Light, Blue, Yellow and Red) and two levels of ARTERIES (RMCA and LMCA). In men, there was no main effect of REGIONS, *P *= NS, but there was a tendency for subcortical peaks to be higher than cortical peaks in men. There was a main effect of STIMULATIONS, *F*(4,24) = 4.2, *MSE *= 11586, *P <*0.01. There was no main effect of ARTERIES, *P *= NS. There was a REGIONS × STIMULATIONS interaction, *F*(4,24) = 4.8, *MSE *= 31945, *P <*0.01. There was no REGIONS × ARTERIES interaction, *P *= NS. There was a STIMULATIONS × ARTERIES interaction, *F*(4,24) = 4.7, *MSE *= 7397, *P <*0.01. There was a three-way interaction, REGIONS × STIMULATIONS × ARTERIES, *F*(4,24) = 4.76, *MSE *= 25392, *P <*0.01. Figures 3(A-D) shows the plot of means of the three-way interaction of the spectral density estimates for all study conditions at **C**-peaks and **S**-peaks in men (Figures 3A-B), and women (Figures 3C-D), respectively.

An analysis of covariance (ANCOVA) with repeated measures was applied to demonstrate that the difference found during color stimulation persists even when the difference due to baseline condition was partialled out. In men, there was significant increase in MFV produced by luminance, hence WAVELENGTH-encoding activity could only be adjudged after partialling out the changes under Dark and Light conditions as changing covariates. Conversely, in women, there was no change in MFV associated with luminance, and hence only baseline Dark condition was used as a covariate.

In men, in the RMCA, at **C**-peaks, there was a tendency for ENERGY-encoding, *F*(1,5) = 5.95, *MSE *= 10315, *P = 0.058*. However, at **S**-peaks, there was a significant main effect of WAVELENGTH-encoding, *F*(1,5) = 9.98, *MSE *= 1016.8, *P < 0.05*. This may suggest that, there was WAVELENGTH-differencing in the right hemisphere in men. There was no luminance effect in the RMCA territory, *P *= NS. However, in the LMCA, there was an orthogonal LUMINANCE effect, *F*(1,5) = 6.27, *MSE *= 6834.6, *P < 0.05*. Figure 3A shows that the RMCA **S**-peaks were 'topologically' separated, achromatic from chromatic peaks, and between the latter, short wavelength (Blue) from long wavelength (Yellow and Red). There was a reverse tendency at the **C**-peaks. Overall, in men, wavelength-differencing in the right hemisphere (Figure 3A), and luminance effect in the left hemisphere (Figure 3B), occurred by processes of CLTD and SLTP.

In women, in the RMCA, the **C**-peaks were unremarkable, *P *= NS. However, at **S**-peaks, there was some residual tendency for ENERGY-encoding effect, *F*(1,6) = 5.65, *MSE *= 31823, *P = 0.054*, and LUMINANCE sensitivity, *F*(1,6) = 5.52, *MSE *= 58620.5, *P = 0.057*. On the other hand, in the LMCA, a main effect for ENERGY-encoding occurred at **C**-peaks, *F*(1,6) = 6.35, *MSE *= 34730, *P < 0.05*, as well as at **S**-peaks, *F*(1,6) = 7.5, *MSE *= 2197.7, *P < 0.05*. This may suggest that in women, FREQUENCY-differencing occurred in the left hemisphere. A parallel LUMINANCE main effect, occurred at **C**-peaks, *F*(1,6) = 6.27, *MSE *= 10461.6, *P < 0.05*, but showed a tendency at **S**-peaks, *F*(1,6) = 5.5, *MSE *= 58620, *P = 0.057*. In women, both frequency-differencing and luminance effect responsiveness in the left hemisphere (Figure 3D), occurred by processes of CLTP and SLTD.

## Discussion

Overall, there were gender-related differences in color processing. The main results demonstrated that, there was right hemisphere wavelength-differencing in men, but left hemisphere frequency-differencing in women. The latter may suggest that, color processing followed the same rule of lateralization as other major cognitive functions, such as facial processing [9,13,15] and general intelligence [14], which implicated the right hemisphere cognitive style in men, and left hemisphere cognitive style in women. The reason for gender-related cerebral asymmetry for color processing remains to be elucidated. However, it may be suggested that, the neuroadaptation to the physical qualities of light as a wave and as quanta energy, pre-conditioned the right hemisphere for wavelength-differencing and left hemisphere for frequency-differencing, respectively.

A light hypothesis for cerebral asymmetry could be postulated, to infer that, the phenotypic neuroadaptation to the environmental physical constraints of light, led to phenotypic evolution and genetic variation of X-Y gene pairs that determined hemispheric asymmetry. The evolutionary trend is towards optimization of perception of the 'whole' environment by functional coupling of the genes for complementarity of both hemispheres within self, and between both genders. The contralateral hemisphere processing of wavelength-differencing and frequency-differencing was as a result of the inverse relationship between wavelength and frequency.

The results further demonstrate that, color processing was organized within cortico-subcortical circuits. It could be presumed that, the neuronal assemblies processing light information as well as their blood flow supply, share analogous topological organization [1,9]. In men, the distribution of the **S**-peaks showed that dark elicited the least effect followed by White, Blue, Yellow and Red (Figures 2 and 3). This type of summation of responses, with distinction between achromatic and chromatic contrasts along orthogonal axis, on one hand, and between short wavelength and long wavelength on the other, could be presumed to be evidence for stimulus complexity topological organization based on wavelength, in the right hemisphere in men. The latter extends from an area implicated in luminance processing to a much greater area for wavelength-differencing. Similar stimulus complexity topological organization, has been observed in studies involving facial and object stimuli in the right hemisphere in men [9,28]. In other words, there is a topological organization, based on category-specific stimulus complexity of the functions, located within the ventral temporal cortex of the right hemisphere in men. The latter would be compatible with the findings that thin stripes in V2 contain functional maps where the color of a stimulus is represented by the location of its response activation peak [29,30]. Conversely, in the left hemisphere in women, at C-peaks (Figures 2 and 3), frequency-differencing separated the peak for high frequency color (Blue) from low frequency color (Yellow), but was parallel to the luminance axis. Hence, the differentiation was process-mapped only to frequency. Similar process-map model has been proposed for facial processing [9,31]. It could be suggested that in the left hemisphere in women, there is a category-specific process-mapping system for retrieving color from memory. The latter would be consonant to the proposed distinct map for representation of color in memory [32,33].

Gender differences in color processing in the present study were observed at the neural processing stage. However, there is indication that there may be receptoral differences, including genetic differences in cone pigments between men and women [34]. The gender differences in color processing mechanisms may have behavioral correlates, for example, the co-localization of color, memory and language processing in the left hemisphere in women, may explain the greater access to a larger repertoire of words to describe colors across ages, languages and cultures, and better ability at color matching from memory [35]. Brain lesion studies in men and women with color deficits, may be applied to further explore gender differences.

The mechanisms underlying vasomotor changes during color processing have not been fully elucidated, but may be inferred from what is known. Cerebral arteries are innervated by postganglionic nitrergic nerves, originating from the ipsilateral pterygopalatine ganglion, that tonically dilate cerebral arteries in the resting condition [36]. The observed changes in MFV in response to visual stimulations have been related to imbalance between sympathetic vasoconstrictor traffic and vasodilator effects of nitric oxide (NO) [19,37]. The NO released postsynaptically, diffuses back across the synaptic cleft, to act on the presynaptic terminals, causing increases in presynaptic glutamate release [36,38], which could account for ipsilateral LTP [10,38]. Others contend that LTP induced in the visual cortex in animal models is NO dependent [38]. The LTP and LTD recorded non-invasively using *f*TCDS, have shown plasticity during color processing over several hours [10], and hence could be applied to the study of stroke rehabilitation and monitoring of drug effects on N-Methyl-D-aspartate (NMDA) receptors.

The results suggest that there were profound gender-related differences in cortico-subcortical activation during color stimulation. In men, in the right hemisphere, SLTP and CLTD occurred with wavelength-differencing. Conversely, in women in the left hemisphere, CLTP and SLTD occurred with frequency-differencing. It is not clear at this time, if these findings would have clinical implications for brain lesions in men and women, respectively. However, one plausible implication of these findings could be that, while in men, subcortical lesions of the right hemisphere may be associated with severe color deficits because of impaired SLTP processes for wavelength-differencing, in women, cortical lesions of the left hemisphere could result in more severe deficits due to inability to form CLTP processes for frequency-differencing.

In conclusion, gender differences in color processing implicated right hemisphere wavelength-differencing in men, but left hemisphere frequency-differencing in women. Future research using *f*TCDS technique should explore clinical applications of color processing in stroke rehabilitation, and monitoring of drug effects. Genetic and comparative animal experiments, as well as brain lesion studies are needed to further elucidate mechanisms of gender differences in color processing.

## Disclosures

The author declares that they have no competing interests.
